# Well-Known and Novel Players in Endothelial Dysfunction: Updates on a Notch(ed) Landscape

**DOI:** 10.3390/biomedicines9080997

**Published:** 2021-08-11

**Authors:** Francesca Fortini, Francesco Vieceli Dalla Sega, Luisa Marracino, Paolo Severi, Claudio Rapezzi, Paola Rizzo, Roberto Ferrari

**Affiliations:** 1 Maria Cecilia Hospital, GVM Care & Research, 48033 Cotignola, Italy; ffortini@gvmnet.it (F.F.); fvieceli@gvmnet.it (F.V.D.S.); rpzcld@unife.it (C.R.); rzzpla@unife.it (P.R.); 2 Laboratory for Technologies of Advanced Therapies (LTTA), Department of Translational Medicine, University of Ferrara, 44121 Ferrara, Italy; mrrlsu@unife.it (L.M.); svrpla@unife.it (P.S.)

**Keywords:** endothelial dysfunction, pyroptosis, Notch, COVID-19, angiotensin converting enzyme inhibitors, angiotensin II receptor blockers, statin, ANGPTL3, ARB, ACEi

## Abstract

Endothelial dysfunction characterizes every aspect of the so-called cardiovascular continuum, a series of events ranging from hypertension to the development of atherosclerosis and, finally, to coronary heart disease, thrombus formation, myocardial infarction, and heart failure. Endothelial dysfunction is the main prognostic factor for the progression of vascular disorders, which responds to drug intervention and lifestyle changes. Virtually all of the drugs used to prevent cardiovascular disorders, such as long-used and new antilipidemic agents and inhibitors of angiotensin enzyme (ACEi), exert an important effect on the endothelium. Endothelial dysfunction is a central feature of coronavirus disease -19 (COVID-19), and it is now clear that life-risk complications of the disease are prompted by alterations of the endothelium induced by viral infection. As a consequence, the progression of COVID-19 is worse in the subjects in whom endothelial dysfunction is already present, such as elderly, diabetic, obese, and hypertensive patients. Importantly, circulating biomarkers of endothelial activation and injury predict the severity and mortality of the disease and can be used to evaluate the efficacy of treatments. The purpose of this review is to provide updates on endothelial function by discussing its clinical relevance in the cardiovascular continuum, the latest insights from molecular and cellular biology, and their implications for clinical practice, with a focus on new actors, such as the Notch signaling and emerging therapies for cardiovascular disease.

## 1. Premise

The average human endothelium weighs 1.5 kg with a surface area of more than 800 m^2^. It constitutes the lining of the vessels and of the heart as a continuous layer of cells, similar to tiles on the floor [1]. The human endothelium is able to produce more than 250 biologically active substances that help regulate vascular structure and function, and it is the bodyguard of vascular health, including many other activities that are not in the scope of this article.

The activity of the endothelium responds to various hormones, neurotransmitters, and vasoactive factors, but the major determinant of endothelium function is laminar shear stress, the tangential force exerted on the endothelium by blood, which causes a positive transcriptional and post-translation control on the activity of the nitric oxide (NO) synthase (the enzyme involved in the synthesis of NO from the amino acid L-arginine) [2]. Laminar shear stress is crucial for the transcription and post-translational modification of a plethora of genes and several proteins, respectively, which are essential to maintain the endothelium in a non-proliferative state, to determine the endothelial cells morphology, to preserve the integrity of the endothelium, and to block the expression of genes coding for proteins that mediate the adhesion of inflammatory cells to the endothelium, such as intercellular adhesion molecule (ICAM)-1 and vascular adhesion molecule (VCAM)-1 [3]. Laminar shear stress is fundamental for the most relevant action of the human endothelium, which is the control of vascular smooth muscle tone (and, therefore, blood pressure) through the secretion of relaxing and contracting factors such as inducers of vasodilation such as NO and prostacyclin 2 (PGI_2_), endothelium-derived relaxing factors (EDRFs), and vasoconstrictors such as endothelin-1 (ET-1) and thromboxane A_2_ (TxA_2_). There is a constant release of several EDRFs, which counteract vasoconstrictor substances, such as noradrenaline, angiotensin II, or ET-1 [4]. The normal functioning endothelium is able to increase the release of EDRFs in response not only to the shear stress exerted by the circulating blood, but also by physiological stimuli such as the humoral stimulation by vasoactive substances such as acetylcholine or bradykinin [5].

Thus, the endothelium is both a target and a modulator of blood pressure-related and hormonal influences [1]. Alterations of endothelial function, such as an impaired release of EDRFs, develop in many pathological conditions, including hypertension and coronary artery disease (CAD).

Endothelial cells also exert a central role in the coagulation process by regulating the expression of anticoagulant and procoagulant factors on the cell surface and, once again, laminar shear stress contributes to this anticoagulatory state of the endothelium by promoting the transcription of Kruppel Like Factor (KLF) 2 and KLF 4 [6]. Insults to the endothelium caused by agents such as oxidized low-density lipoproteins (oxLDL), inflammatory cytokines, hypoxia, and lack of estrogens lead to a decrease of NO synthesis, an increase of neointimal thickness, over-expression of ICAM-1 and VCAM-1, induction of a procoagulatory state, and increased apoptosis [7]. This, in turn, is responsible for interruption of endothelium integrity leading to lipids and macrophage infiltration in the sub-intimal space, thus providing for atherosclerotic plaque formation. Disturbed, turbulent blood flow present in regions of the arteries characterized by bends and curvature (such as the inner curvature of the aortic arch) is unable to induce the “protective” gene expression profile and is associated with increased permeability and chronic low inflammation, predisposing the endothelium to be more susceptible to those risk factors that favor the emergence of atherosclerosis lesion [8,9].

Emerging evidence in multiple research fields, including distant from the cardiovascular, are contributing to the discovery of novel markers of endothelial dysfunction. For example, studies of developmental biology have identified pathways, such as the Notch [8,10,11], Wnt [12], Hedgehog [13], and Hippo [14], that play a major role not only during the embryonic development of the cardiovascular system but also post-natally in the maintenance of endothelium integrity and function. Among them, the Notch pathway is a primary transducer of the positive effect of shear stress on the endothelium [8]. Additionally, studies in COVID-19 patients have shown that endothelial dysfunction is involved in pneumonia and the multiorgan damage caused by SARS-CoV-2 infection and are shedding light on the molecular details underlying the endothelial damages caused by dysregulated inflammation [15].

The aim of this review is to provide updates on endothelial dysfunction, with particular attention to the emerging role of the Notch pathway, in the context of existing and novel approaches for treatment and prevention of cardiovascular diseases, including the complications of COVID-19.

## 2. Endothelium as a Barometer of Cardiovascular Disease

Endothelial function is considered a barometer for cardiovascular risks. Endothelium dysfunction is instrumental in every step of the so-called cardiovascular continuum, a series of events that range from hypertension to the development of atherosclerosis and, eventually, to CAD, thrombus formation, myocardial infarction, and heart failure [16]. Dysfunction of the endothelium represents the major prognostic factor of the progression of vascular disorders, which responds to pharmacological intervention and lifestyle modifications [16]. Both angiotensin-converting enzyme inhibitors (ACEi) and lipid lowering agents have been shown to reduce the progression of atherosclerosis and improve the prognosis of CAD patients [17,18]. However, deeper knowledge of the precise molecular mechanisms underlying the endothelial dysfunction may lead to the identification of novel targets for those patients not responding to existing therapies. Similarly, since there is no single ideal method for measurement of endothelial function in humans, future studies will lead to the identification of biomarkers that can be integrated with existing measurements of a patient-specific determination of endothelial function, thus improving the stratification of patients for differential diagnosis, disease progression, and responses to therapy [16].

### 2.1. The Importance of Notch Signaling in the Endothelium

The Notch signaling, a mediator of communication between adjacent cells, regulates endothelial cells identity and function during embryonic development and throughout life [19]. In mammals, Notch signaling comprises receptors (Notch 1–4) and ligands (Delta-like 1, 3, 4 and Jagged 1, 2) present on the surface of adjacent cells. The interaction between receptors and ligands is required for the activation of Notch, which requires two proteolytic cuts that release the active form of Notch, which translocates into the nucleus and acts as a regulator of transcription [20]. The role of the Notch pathway as a master regulator of angiogenesis is well established [19], and there is now accumulating evidence that the Notch signaling plays a more important role by transducing the signals provided by the blood shear stress to the endothelium [21,22]. Active Notch confers a pro-survival, anti-inflammatory, and anti-atherogenic environment, contrasting endothelial apoptosis induced by inflammatory cytokines, such as tumor necrosis factor (TNF)-α and interleukin (IL)-1β [23,24,25], reducing the expression of adhesion molecules such as ICAM-1 and VCAM-1 [26], and maintaining the integrity of the endothelium by participating in the formation of the endothelial junction complex [27]. Notch is inhibited by known cardiovascular risk factors causing endothelial dysfunction, such as oxidative stress [28], dyslipidemia [29], and low levels of estrogens [24,30]. It is therefore possible to envision the development of novel therapeutic strategies against endothelial dysfunction based on re-establishing the physiological levels of Notch in this tissue. Circulating levels of the Notch pathway components can represent novel biomarkers, providing information on the status of the endothelium, similarly to endothelial extracellular vesicles (EVs) and endothelial progenitor cells (EPCs) [16]. Circulating levels of Notch ligands have been found to be associated with the progression of heart failure [31]. A multitude of studies has shown that the excessive inflammatory response against the severe acute respiratory syndrome coronavirus 2 (SARS-CoV-2) infection causes massive endothelium damage, and as discussed more thoroughly in the following paragraphs, it is well established that endothelial dysfunction underlies the multiorgan damage caused by SARS-CoV-2 in patients with COVID-19. Notch is involved in multiple steps of the progression of COVID-19, beginning with entrance of the virus into the cell and the consequent inflammatory response leading to endothelium apoptosis and its thrombogenic status [32,33]. Several experimental data exist supporting the involvement of Notch in endothelial dysfunction caused by COVID-19. In a Rhesus macaque model of SARS-CoV-2 infection, the transcriptional signatures induced by the virus revealed an alteration of the Notch signaling in the lungs of the macaques [34]. Furthermore, computational modeling of host protein interactions with SARS-CoV-2 showed that proteins interacting with the 5′-region of SARS-CoV-2 RNA are functionally related to Notch2 [35]. Finally, the discovery in COVID-19 patients of an association between increased Notch4 expression on circulating regulatory T (Treg) cells and disease severity has important therapeutic implications for this pathology [36].

### 2.2. The Importance of the Endothelium Continuity

Independently from their biological function and possible markers, endothelial cells are important, as they constitute a physiological barrier against atherosclerosis formation and progression. The maintenance of endothelium continuity is at the basis of the so-called pleiotropic effects of preventative therapy for cardiovascular diseases with ACEi and statins, which strictly depend on the life and death of the endothelium.

The endothelium undergoes a life and death cycle involving the process of programmed cell suicide or apoptosis [37,38], matched by a subsequent regeneration. Apoptosis, which should be distinguished from necrosis, is a “physiological” form of cell death, which originates in the nuclei, occurs in one cell at a time, and does not evoke an immunological response. It is accompanied by renewal and regeneration. Thus, the entire endothelium of the human body is continuously regenerated, with a lifespan of about 1 to 3 months [1]. Mature endothelial cells possess limited regenerative capacity, but the bone marrow produces endothelial progenitor cells (EPCs), which are able to locate the site of the injury or damage and may play a role in angiogenesis and contribute to the maintenance of the endothelial layer.

If there is an imbalance between the endothelial life (regeneration) and death (apoptosis) cycle, with apoptosis outweighing regeneration, then there is a loss of continuity of the vessels wall, thus favoring the occurrence and progression of atherosclerosis [1,38]. An imbalance in cellular apoptosis and regeneration of the endothelium covering an atherosclerotic plaque can lead to thrombus formation and to acute coronary syndromes (ACS). EPCs can be determined in the blood while endothelial apoptosis can be measured by incubating human umbilical vein endothelial cells (HUVECs), or other human endothelial cells, with serum from normal individuals or patients with CAD of different severities or different pathologies [39,40]. Using this method, we determined the rate of endothelial apoptosis in patients with proven CAD. We showed that serum from patients with chronic coronary syndrome, with or without angina, induces a significant increase in the rate of apoptosis versus serum from healthy age-matched controls, and the ratio of BAX/Bcl-2, the two proteins that control apoptosis, was also increased, suggesting that serum from stable CAD patients includes agents that promote endothelial apoptosis [40]. Moreover, serum from patients suffering an acute coronary syndrome, mainly an ST elevation myocardial infarction, further increases the rate of endothelial apoptosis [40]. This is associated with a concomitant stepwise (from volunteers to patients with stable CAD and those with ACS) reduction of the eNOS expression, an established marker of endothelial function [41]. Our previous data using the same methodology suggest that serum from patients with severe heart failure shares a rate of apoptosis similar or superior to one of patients with ACS [39].

Another way to evaluate endothelial dysfunction in humans is the measurement of certain “classical” indicators, for example the pro-coagulant von Willebrand Factor (vWF) or by the evaluation of endothelial-dependent dilatation or ischemia-induced flow-mediated dilatation. This last technique has been popular for testing several anti-hypertensive drugs, such as calcium-antagonists, ACE inhibitors, and angiotensin II blockers (ARBs). However, only two classes of drugs thus far have been carefully evaluated for their effects on the endothelium life and death cycle. These are ACE inhibitors and statins.

## 3. Focus on Ace Inhibitors (ACEi)

**Figure 1:**
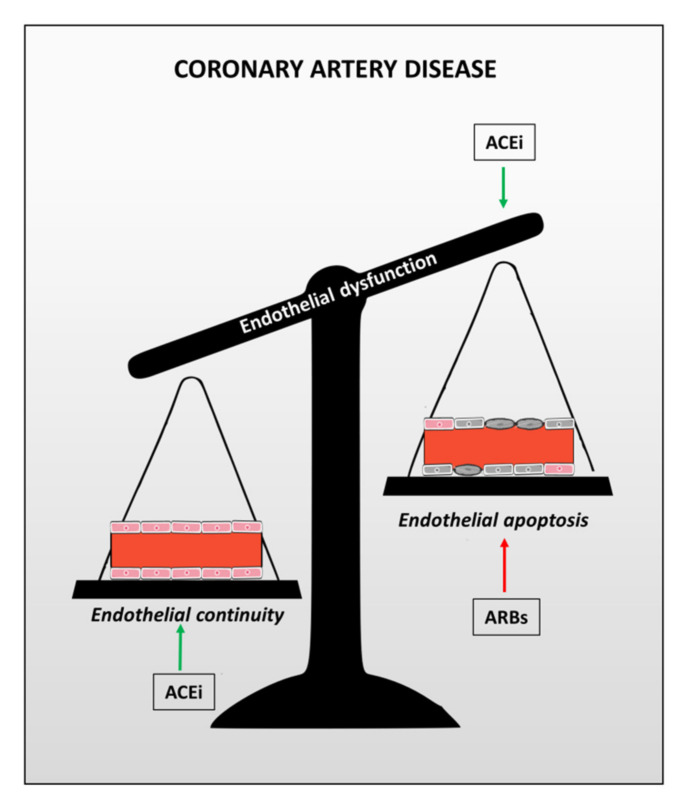
Opposite effects of ACE inhibitors (ACEi) and angiotensin II receptor blockers (ARBs) on endothelial function. Conversely, ACE inhibition reduces cell death (apoptosis) and extends the lifecycle of endothelial cells. Thus, these drugs maintain endothelial continuity and exert protection against acute coronary syndromes and cardiovascular mortality beyond blood pressure reduction. However, ARBs appear to exert a pro-apoptotic effect on vascular endothelial cells.

ACE inhibitors are widely used to counteract the entire “cardiovascular continuum” by reducing blood pressure, to angina, by slowing down the progression of coronary atherosclerosis, to myocardial infarction and heart failure, by reducing the progression of left ventricular remodeling. These effects are independent of blood pressure reduction and the reason ACEi can be beneficial to the coronary arteries beyond blood pressure reduction became clear after translational studies, revealing that ACE inhibition reduces endothelial apoptosis and increases the production of EPCs, with a positive effect on both the beginning and the end of the endothelial cell lifecycle [37,42]. Thus, ACEi maintain endothelial continuity and reduce the progression of coronary atherosclerosis and atherothrombosis (Figure 1). However, only the active metabolites of ramipril (ramiprilat) and perindopril (perindoprilat), but not trandolapril (trandolaprilat) or quinapril (quinaprilat), have been shown to reduce human endothelial apoptosis. In clinical trials, only perindopril in EUROPE and ramipril in HOPE improved prognosis, while trandolapril in PEACE and quinapril in QUIET did not. Other studies demonstrate that both ramipril and perindopril have a high affinity for vascular ACE (the enzyme targeted in CAD), with the strongest affinity for bradykinin binding sites. This binding results in a powerful reduction of the breakdown of bradykinin, which is pivotal to coronary protection. Thus, both a plausible mechanistic reason and solid clinical data exist for the use of ACEi in patients with stable CAD behind blood pressure reduction.

## 4. Focus on Angiotensin II Receptor Blockers (ARBs)

**Figure 2:**
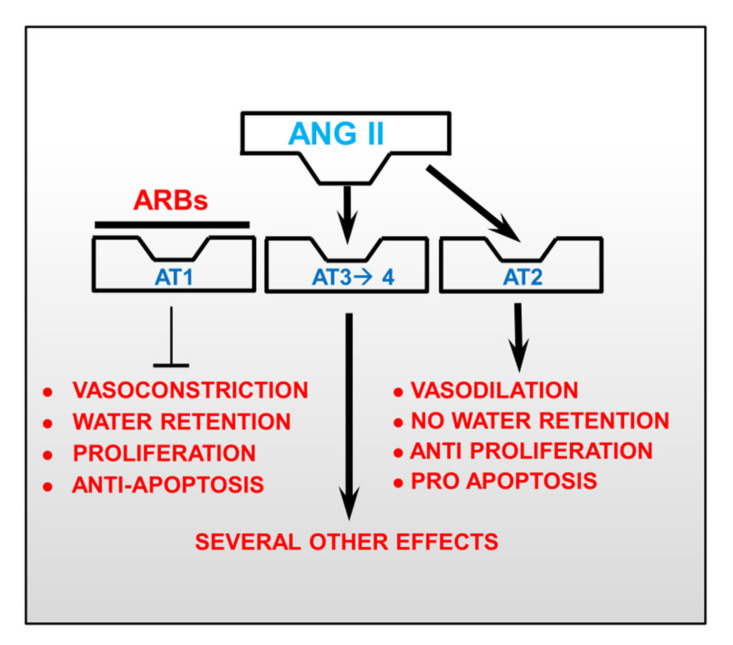
Effects of angiotensin II receptor blockers (ARBs). ARBs bind to angiotensin II (Ang II) receptor type 1 (AT1) contrasting vasoconstriction, water retention, promoting proliferation, and limiting apoptosis in the endothelium. When AT1 is inhibited, the circulating Ang II can only bind to the Ang II receptor type 2 (AT2) causing vasodilation, no water retention, reduced proliferation, and increased apoptosis of endothelial cells. In addition, Ang II may also bind to Ang II receptor type 3 and 4 (AT3 and AT4), exerting additional effects that are not fully known yet.

The aforementioned anti-apoptotic effect of ACEi is not shared by the other classes of renin-angiotensin inhibitors: the angiotensin II receptor blockers (ARBs) (Figure 1). In 2006, from trials on hypertension, ARBs do not improve CV (cardiovascular) outcome and may increase the occurrence of myocardial infarction: a phenomenon called “myocardial paradox of ARBs” [43]. This caused a “tempest” within the medical community, and consequently, several meta-analyses supported the concept that ARBs are less CV protective than ACEi [44,45,46,47,48]. Nevertheless, the debate continued with different and opposite viewpoints [49,50]. Several individual trials do not show the cardioprotective effects of ARBs, while there is clear positive evidence for ACEi. The reason for this is not clear, and the explanation, once again, is related to apoptosis of the endothelium. A study compared the effects of perindopril vs valsartan in patients several hours after having suffered an ST-elevated myocardial infarction. As expected, perindopril reduced the pro-apoptotic effect of the serum and increased the mobilization of bone-marrow cells; however, valsartan did not [37]. It seems that valsartan favored the pro-apoptotic effect of the serum [37]. This may be the consequence of a receptorial shift. As shown in Figure 2, ARBs inhibit the angiotensin receptor type 1 (AT1), which is responsible for the anti-hypertensive action of ARBs. However, as a result of this inhibition, the circulating angiotensin II will now preferentially bind to the angiotensin receptor type 2 (AT2), which, unfortunately, has a pro-apoptotic action.

## 5. Focus on Lipid Lowering Agents

**Figure 3:**
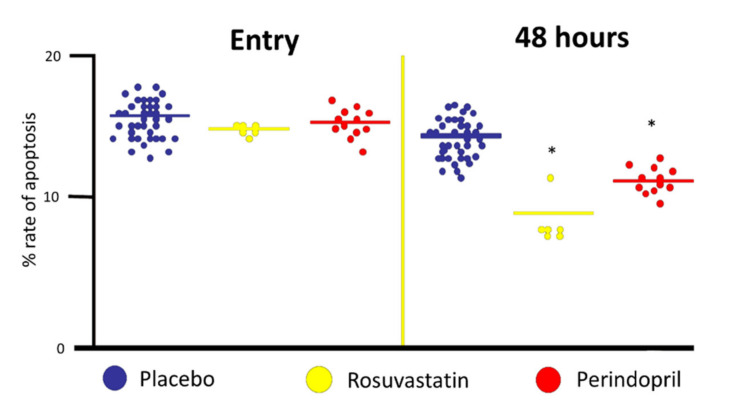
Effects of 48 h incubation of human endothelial umbilical cells (HUVECs) with serum from patients with acute ST-elevation myocardial infarction at entry (within 3 h from symptoms) and 48 h later. Patients were treated with either rosuvastatin 20 mg or perindopril 10 mg. * *p* < 0.05, different from controls.

The use of statins to prevent and treat coronary artery diseases is universally recognized, and it is well accepted that their beneficial effects extend beyond cholesterol reduction, the so-called pleiotropic effects. Among the various explanations for these “unconventional” effects, one of the most accredited is that, similarly to ACEi, statins interfere with the life and death cycle of the endothelium. It is known that statins inhibit the pro-caspase 9, which, in turn, has a pro-apoptotic effect, and rosuvastatin has been shown to increase circulation bone-marrow-derived EPCs, enhance vascular re-endothelialization, and reduce neo-intimal formation in a model of mice carotid artery injury. Studies conducted by us investigated the effects of both perindopril and rosuvastatin on the rate of apoptosis of patients affected by a myocardial infarction at entry (within one hour) and 48 h afterward. Serum from patients who received either perindopril or rosuvastatin shows an important reduction of the rate of apoptosis (unpublished data (Figure 3), and [51,52]). Together with aspirin, every guideline recommends, as preventative therapy for cardiovascular disease, ACEi and statins, independently from blood pressure and cholesterol levels and the maintenance of endothelium continuity could be the reason for their beneficial action proved in several clinical trials. Of interest, statins have been shown to activate Notch1 signaling in the endothelium [53].

## 6. Focus on Angiopoietin-like Proteins (ANGPTL3) Inhibitors

Angiopoietin-like proteins (ANGPTLs) are important regulators of lipoprotein metabolism that have emerged as a promising molecular target for modulation of lipid levels and cardiovascular risk. Evinacumab, a recombinant monoclonal antibody against human ANGPTL3, has been recently approved by the FDA for the treatment of homozygous familial hypercholesterolemia (HoFH), and other ANGPTL3 inhibitors (including antisense oligonucleotide, ASO) are currently at different stages of clinical trials. Individuals bearing loss-of-function mutations of the ANGPTL3 gene exhibit familial hypolipidemia and those with complete ANGPTL3 deficiency do not display signs of coronary atherosclerosis [54]. Decreased ANGPTL3 activity results in lower levels of triglycerides (TG), LDL cholesterol, and high-density lipoproteins (HDL) cholesterol as shown in family and general population studies [55]. The effect of ANGPTL3 inhibition has been explained in terms of modulation of lipid traits but an effect on endothelium has also been proposed. In apoE*3Leiden.CETP mice, a well-established humanized model characterized by elevated TG and very-low-density lipoproteins (VLDL) cholesterol, treatment with evinacumab reduced the expression of ICAM-1, the adhesion on monocytes to the endothelium, and the number of macrophages infiltrating the plaque [56]. In diabetic mice fed a high-fat diet, the hypoglycemic drug vildagliptin reduced circulating glucose, together with LDL cholesterol and ANGPTL3 expression in the aorta, and improved endothelial function [57].

**Figure 4:**
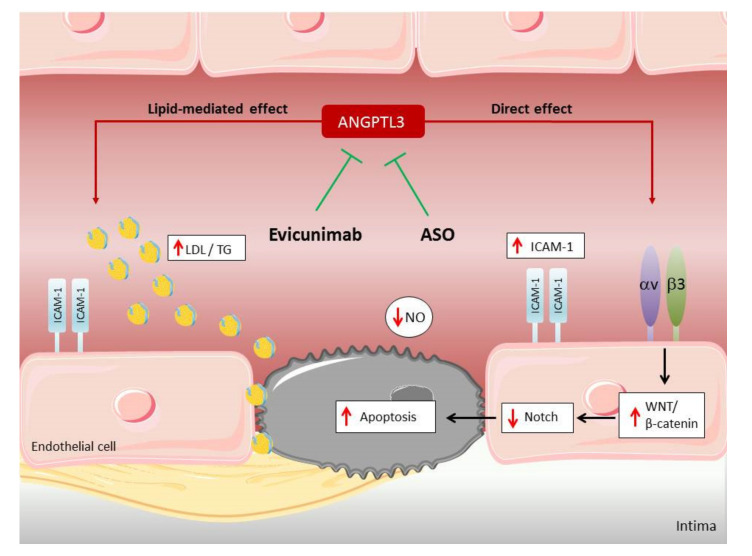
Lipid-mediated and possible direct effects of ANGPTL3 inhibitors on endothelial function. Endothelial dysfunction (ED) is characterized by increased apoptosis, expression of intercellular adhesion molecule (ICAM-1), and impairment of nitric oxide (NO) synthesis. ANGPTL3 inhibitors, such as the antibody Evinacumab and antisense oligonucleotide (ASO), reduce circulating low density lipoproteins (LDL) and triglycerides (TG), hence contrasting lipid-mediated ED. In addition, ANGPTL3 inhibitors may directly protect from ED by impeding the binding of ANGPTL3 to endothelial integrin αvβ3, thus inhibiting the Wnt/β-catenin signaling and restoring protective Notch signaling pathway.

The improvement of endothelial function exerted by ANGPTL3 inhibitors may be explained by decreased circulating TG and LDL, and therefore less infiltration in the endothelium, but ANGPTL3, beyond its effect on lipids, seems to affect endothelial dysfunction directly by binding endothelial integrin αvβ3 inducing endothelial cell adhesion and migration [58] and by stimulating the Wnt/β-catenin signaling, constituted of transmembrane receptors Frizzled and several Wnt ligand proteins [12]. The binding of Wnt to Frizzled allows β-catenin to translocate to the nucleus, where it controls gene transcription related to endothelial function [12]. β-catenin also stabilizes endothelial cell-to-cell adhesion by modulating vascular endothelial cadherin and N-cadherin through activation of phosphatidylinositol3-kinase (PI3K)/Akt/Glycogen synthase kinase-3 beta (GSK3β) pathway [59] which, in turn, is an inhibitor of the Notch signaling [60], a major regulator of angiogenesis and required for the correct functioning of the endothelium [10,20]. The few existing studies in endothelial cells show an effect of ANGPTL3 on cell adhesion and angiogenesis [58], providing support to the hypothesis that, in addition to the effect on lipids, ANGPTL3 has a direct impact on endothelial cells, and likely on atherosclerosis, of which neo-angiogenesis is an important hallmark [61]. Lipid-mediated and possible direct effects of ANGPTL3 inhibitors on endothelial function are summarized in Figure 4.

## 7. Importance of Endothelial Dysfunction in the COVID-19 Pneumonia

The majority of COVID-19 cases is asymptomatic or exhibits mild to moderate symptoms, but approximately 20% of patients develops severe illness characterized by atypical interstitial bilateral pneumonia that can progress to acute respiratory distress syndrome (ARDS) and multiple organ failure [62]. COVID-19-induced ARDS consists of two phenotypes: the alveolar and the vascular type, the latter being more prominent and aggressive. The vascular phenotype is a vascular disease that primarily involves the endothelium, thus explaining the worse progression of COVID-19 in elderly, diabetic, obese, and patients with hypertension in which endothelial dysfunction is already present to some degree. Thus, it is now clear that in the pathogenesis of COVID-19, the endothelium is located at the crossroad between inflammation, of which it is the target organ, and coagulation, of which is a key regulator [15]. The involvement of the endothelium can be the result of both the direct infection from the virus and an indirect, uncontrolled, virus-mediated host immune response.

### 7.1. Focus on Direct Endothelium Infection by SARS-CoV-2

SARS-CoV-2 enters into the host cell mainly through binding of the viral spike (S) glycoprotein to the angiotensin-converting enzyme 2 (ACE2) receptor, followed by proteolytic cleavage mediated by human proteases, such as transmembrane serine protease 2 (TMPRSS2). It has also been shown that cell entry of SARS-CoV-2 is pre-activated by proprotein convertase furin [63]. ACE2 is expressed by both pneumocytes and endothelial cells [64]; hence, the direct infection of endothelial cells appears plausible but whether it happens is still under debate. Evidence of the ability of the SARS-CoV-2 to infect endothelial cells has been provided by Monteil et al., who have shown that the virus can infect engineered human blood vessel organoids [65]. Transmission electron microscopy analysis of lungs, kidneys, heart, and small intestine of patients who died from COVID-19 revealed the presence of the virus in the endothelial cells [66,67]. Histological analyses of tissues have shown endothelial damage, characterized by mononuclear cells infiltrate and loss of endothelial integrity due to the disruption of intercellular junctions, cell swelling, loss of contact with the basal membrane [67], and widespread thrombosis in the lung microvasculature [66]. Several other electron microscopy analyses have confirmed the presence of virus-like particles in endothelial cells [68,69]. Recently, however, the interpretation of electron microscopy images has been disputed, suggesting that the viral-like particles may be cytoplasmic structures [70,71]. Other histologic examinations of post-mortem lungs failed to show evidence of endothelial cell infection [72], and a pre-print study showed that aortic, microvascular, and blood outgrowth endothelial cells are resistant to in vitro infection with SARS-CoV-2 [73]. Strong evidence of infection and virus production in the endothelium of mice and non-human primates infected with SARS-CoV-2 have been recently obtained [74].

### 7.2. Focus on the Endothelium Inflammation Caused by SARS-CoV-2

SARS-CoV-2 infection causes the hyperactivation of the immune response of the host, characterized by an unrestrained activation of the complement system and an uncontrolled release of pro-inflammatory molecules, such as IL-6, IL-1, and TNF-α (referred to as cytokine storm) by macrophages. This hyperinflammatory state contributes to endothelial dysfunction, and the damaged endothelium itself is a source of inflammatory mediators contributing to the amplification loop that feeds the cytokine storm [75]. This process is responsible for endothelial cell death, loss of vascular barrier integrity, and tissue damage. The indirect effect of SARS-CoV-2 infection has been confirmed by an in vitro study, in which treatment of human pulmonary microvascular endothelial cells with plasma derived from patients with severe COVID-19 triggered reduced cell viability [76]. Besides activating the vascular endothelium, the cytokine storm also leads to massive platelets activation, contributing to coagulopathy, a well-established clinical manifestation of COVID-19 [77,78]. In COVID-19 patients, the endothelium also presents an increased expression of mediators of thrombosis and leukocytes recruitment [79], such as integrins and selectins, and adhesive proteins, such as vWF and fibrinogen, determining an extensive platelets binding and fibrin formation that leads to thrombosis and disseminated intravascular coagulation (DIC) [62]. Additionally, the activated phenotype of endothelial cells promotes infiltration of leukocytes, in particular neutrophils, which produce neutrophils extracellular traps (NETs), involved in triggering the coagulation cascade [80]. NETs have also been recently found in arterial microthrombi of post-mortem lung tissues of COVID-19 patients [81] and it has been suggested that NETs binding to vWF provide a scaffold for platelet adhesion and thrombus formation [82].

### 7.3. Focus on Biomarkers of Endothelial Function in COVID-19

**Figure 5:**
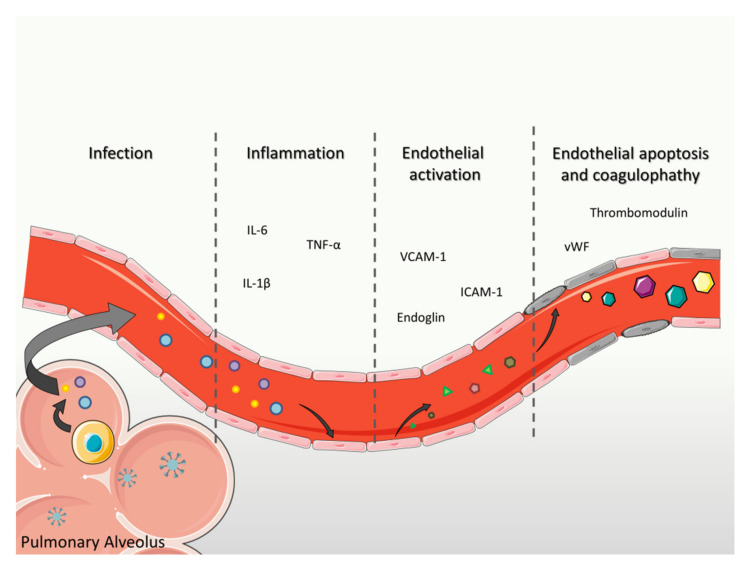
Temporal evolution of endothelial biomarkers in COVID-19. Increase of circulating inflammatory cytokines in response to viral infection induces alteration of endothelial cells that can be detected in term of circulating biomarkers. Early-stage inflammation is characterized by increased interleukin (IL)-1β, tumor necrosis factor (TNF)-α, and IL-6; endothelium releases vascular adhesion molecule (VCAM)-1, intercellular adhesion molecule (ICAM)-1, and endoglin in response to inflammation; damaged endothelium releases mediators of coagulation such as thrombomodulin and von Willebrand Factor (vWF).

Given the central role of the endothelium in the pathophysiology of COVID-19 [15,79], tremendous efforts are being allocated to discover novel biomarkers of COVID-19-mediated endothelial injury to identify prognostic factors able to predict severity and mortality of COVID-19. Increased circulating levels of soluble forms of adhesion molecules, such as endoglin, VCAM-1, and ICAM-1 [83,84] as well as biomarkers of NO impairment, such as L-arginine and asymmetric dimethylarginine (ADMA) [77], and markers of endothelial activation, such as thrombomodulin, P-selectin, vWF [85], and angiopoietin-2 [84,86] have all been reported in COVID-19 patients and have been individually associated to the severity and mortality of COVID-19. The circulating levels of these endothelial biomarkers have a temporal evolution, changing during the progression of the disease. Specifically, patients who will die compared to those who will recover already have higher levels of VCAM-1, ICAM-1, and endoglin at the time of admission, suggesting that the activation of the endothelium occurs in a relatively early phase of the disease. Conversely, plasma levels of thrombomodulin and vWF are similar at admission but increase over time only in non-survivors [83]. The temporal evolution of endothelial biomarkers in COVID-19 is represented in Figure 5. Additionally, COVID-19 patients also have high numbers of circulating endothelial cells, which are positively related to inflammatory cytokines and severity of the disease [87], confirming that endothelial damage characterizes COVID-19.

### 7.4. Focus on Endothelial Apoptosis in COVID-19 Patients

As previously discussed, one of the hallmarks of endothelial dysfunction is apoptotic cells death, which can occur from the direct or indirect effect of SARS-CoV-2. Our previous data have shown that an increase of circulating inflammatory cytokines induces endothelial cells apoptosis [39,40,88]. Recent evidence suggests that inflammatory mediators released in the presence of SARS-CoV-2 infection cause a type of endothelial cell death called pyroptosis [89]. In COVID-19, pyroptosis may be activated by the hyperinflammatory status, inducing the NLRP3 (nucleotide-binding domain, leucine-rich–containing family, pyrin domain–containing-3) inflammasome, which, in turn, leads to caspase-dependent release of the pro-inflammatory cytokines (IL-1β, IL-6, and IL-18) and gasdermin D, the effector protein of pyroptosis [90]. SARS-CoV-2 infection activates NLRP3 inflammasome in COVID-19 patients, and this activation is associated with the severity of COVID-19 [91]. Previous studies have shown that endothelial cell pyroptosis plays a crucial role in endothelial damage that characterizes Kawasaki disease [92] and ARDS [93], which are also two well-established clinical manifestations of COVID-19. These findings, together with the increase of circulating cytokines detected during COVID-19 progression, suggest that the loss of endothelial integrity observed in COVID-19 may be associated with pyroptosis. However, further studies are needed to confirm this hypothesis. Preliminary data obtained in our laboratory have shown that the levels of apoptosis of HUVECs treated with serum from COVID-19 patients are significantly higher compared with serum from patients with severe respiratory failure but negative to SARS-CoV-2 (unpublished data). It will be interesting to investigate whether inhibiting inflammasome or pyroptosis effectors will also reduce endothelial apoptosis in this setting.

## 8. Conclusions

We have performed a long journey in understanding the importance of the endothelium as an essential organ to maintain vascular tone and permeability. This is the good part of the journey. However, the endothelium, when not physiologically functional, can exert bad effects and be instrumental in the pathophysiology of several diseases, including CAD, and COVID-19 caused by the recent pandemic created by SARS-CoV-2 infection.

New insights from molecular and cellular biology studies have shown that the functionality of endothelium is determined by crucial pathways, which are dysregulated in disease. Among them, the Notch pathway controls multiple aspects of endothelial cell biology impaired in cardiovascular diseases that may be re-established by targeting endothelial Notch. Given the importance of this pathway, the effect on Notch of novel therapeutic approaches against endothelial dysfunction, such as ANGPTL3, will need to be carefully investigated. Similarly, studies aimed to identify the specific role of Notch in the endothelial dysfunction associated with SARS-CoV-2 infection may lead to new therapeutic approaches for COVID-19.

Endothelial dysfunction predicts the severity of cardiovascular diseases, and drugs and lifestyle changes can reverse it. It follows that the monitoring of the endothelial function can inform us of the efficacy of therapeutic interventions. However, the assessment of endothelial dysfunction in the clinical setting can be technically challenging and not always reliable. Hopefully, increasing knowledge will allow the discovery of more reliable, not invasive, markers of endothelial function. As discussed in this review, assessing the expression levels of members of the Notch family seems to be a promising approach.

In conclusion, many existing drugs improve the function of the endothelium and are in use by millions of patients at risk from or with CAD. Due to the progress in understanding of cellular and molecular biology of the endothelium, other drugs will be available for patients who respond poorly to existing treatments.
